# LPS-Stimulated Whole Blood Cytokine Production Is Not Related to Disease Behavior in Patients with Quiescent Crohn's Disease

**DOI:** 10.1371/journal.pone.0133932

**Published:** 2015-07-24

**Authors:** Mark M. T. J. Broekman, Hennie M. J. Roelofs, Frank Hoentjen, Renske Wiegertjes, Nicole Stoel, Leo A. Joosten, Dirk J. de Jong, Geert J. A. Wanten

**Affiliations:** 1 Department of Gastroenterology & Hepatology, Radboud University Nijmegen Medical Center, Nijmegen, the Netherlands; 2 Department of Internal Medicine, Radboud University Medical Center, Nijmegen, the Netherlands; Charité, Campus Benjamin Franklin, GERMANY

## Abstract

**Introduction:**

Crohn’s disease (CD) is a chronic inflammatory disease in which cytokines play a pivotal role in the induction and maintenance of inflammation. Innate cytokine production is genetically determined and varies largely between individuals; this might impact the severity of inflammation. We aimed to assess whether ex-vivo endotoxin-stimulated levels of cytokines could be associated with disease phenotype.

**Methods:**

Patients with quiescent CD (Harvey-Bradshaw Index ≤ 4 and negative inflammation markers) who were not using immunomodulating drugs or biologicals were eligible. Historical disease characteristics (localization, behavior, number of bowel resections, drug history, extra-intestinal symptoms) were extracted from medical records. We measured cytokine levels (tumor necrosis factor (TNF)-α, interleukin (IL)-1β, IL-6 and IL-10) in supernatants of lipopolysaccharide (LPS) -stimulated whole blood cultures and correlated these with disease characteristics and age- and sex-matched healthy controls. In addition, we analyzed whether single nucleotide polymorphisms (SNPs) in the promoter region of the *TNF-α* gene were related to TNF-α levels.

**Results:**

We included 75 patients with CD and 24 healthy controls. Six patients were excluded because of increased inflammation markers resulting in a total of 69 patients. The mean age (SD) of patients with CD was 51.2 (12.3) years with a mean (SD) disease duration of 24.1 (11.5) years. Disease localization, peri-anal involvement and behavior were not related to LPS-stimulated TNF-α, IL-1β, IL-6 or IL-10 levels. In addition, combination of localization with behavior to differentiate mild from severe disease type showed no significant differences. TNF-α levels were higher in patients with CD (428 pg/ml IQR [267-468]) compared to healthy controls (459 pg/ml IQR [364-570], p=0.02). We found no associations between SNPs in the promoter region and TNF-α levels.

**Conclusion:**

In this study, innate cytokine production of TNF-α, IL-1β, IL-6 and IL-10 was not related to historical disease characteristics or disease severity in patients with quiescent CD. These findings suggest that genetically determined levels of these cytokines obtained from LPS-stimulated whole blood cultures are not linked with disease behavior or severity.

## Introduction

There is a wide variation in the disease course in patients with Crohn’s Disease (CD) ranging from mild ileal inflammation to involvement of the entire gastro-intestinal tract with complications from penetrating disease and extra-intestinal manifestations. So far no reliable biomarkers have been identified as tools to predict disease course [1]. Among the studied biomarkers, patterns in cytokine production remain of particular interest, because of their significant role in the pathogenesis and course of CD [2–5]. The latter is illustrated by genome wide association studies showing that many susceptibility loci for CD are involved in the regulation of cytokine production [6].

The quantity of cytokines produced by individuals after exposure to a stimulus such as lipopolysaccharide (LPS) varies to a large extent [7,8]. A persons’ cytokine production appears to be genetically regulated for 60–70% [9]. This, in combination with a limited intrapersonal variability makes that individuals can be categorized as high- or low producers [10–13]. Prior research in other inflammation-driven diseases showed an association between innate cytokine production and both disease course and response to treatment [14–16].

In CD the defective mucosal barrier of the gut lumen is predominantly exposed to gram-negative bacteria, containing high levels of LPS. In this way individual differences in cytokine production might steer the severity of inflammation and predict the disease course of patients with CD. With this hypothesis previous studies found an association between innate cytokine production in mucosal tissue and disease behavior. For example, mucosal levels of the pro-inflammatory cytokines tumor necrosis factor (TNF)-α, interleukin (IL)-1β and IL-6 were associated with disease relapse [17,18], while lower mucosal levels of the anti-inflammatory IL-10 increased the risk of disease [19]. However, the feasibility of mucosal cytokine measurements for clinical practice remains limited due to the need for biopsies.

Recent studies with peripheral blood exploring cytokine production of stimulated white blood cells also showed an association with disease phenotype [20,21]. These data suggest that individual cytokine production from peripheral blood might be used as biomarker to stratify patients according to the disease course [1]. An important issue is that most of these studies included patients with either active disease or patients with maintenance therapy, both are known to influence the host cytokine production [16,22–25]. By studying patients with CD currently in remission and not taking immunomodulators or biologicals, we aimed to reduce confounders that modulate cytokine production. For this reason, we measured LPS-stimulated cytokine production of TNF-α and other key cytokines (IL-1β, IL-6 and IL-10) in patients with quiescent CD, not using immunomodulators or biologicals and correlated these data to disease characteristics and phenotype to establish their role in disease development.

## Methods

### Ethics Statement

The local ethics committee, METC Arnhem Nijmegen, the Netherlands, approved the study under the protocol number 2013/091 and written informed consent was obtained from all participating patients. The study complies with the declaration of Helsinki.

### Study population

Patients with CD in remission (defined as a Harvey-Bradshaw Index ≤ 4) were eligible for participation. Patients using non-steroidal anti-inflammatory drugs, biologicals (anti-TNF-α agents) or immunomodulating drugs (thiopurines, glucocorticoids, methotrexate and cyclosporine) other than 5-aminosalicylic derivates in the past six months were not eligible for study participation as these drugs modulate *ex vivo* cytokine production [16,21–24,26–30]. To prevent the formation of a non-representative sample of patients with only mild disease, a history of CD-related abdominal surgery was set as an additional inclusion criterion. Exclusion criteria were concomitant systemic diseases, active malignancy or smoking of more than five cigarettes per day [31]. At the day of inclusion heparinized blood (10 ml) was collected for cytokine measurements together with samples for C-reactive protein (CRP) and hemocytometry (as measured in the central hospital laboratory). Patients with CRP levels >10 mg/l or leukocyte counts outside normal limits were excluded for analysis [32]. Age- and sex-matched healthy controls were included. Patients were enrolled at the Radboud University Medical Center in Nijmegen, the Netherlands.

### Disease phenotype

Medical records were checked prior to cytokine measurements by one investigator (MB) for the following variables: disease duration, disease behavior according to the Montreal classification [33], occurrence of extra- intestinal symptoms, number of bowel resections and the time frame during which glucocorticoids, thiopurines, biologicals, methotrexate or cyclosporine had been prescribed. Besides analyses based on phenotypes derived from the Montreal classification we compared patients with a combination of ileal disease in the absence of fistulas (classified as mild disease type) and patients with penetrating disease in combination with activity in both ileum and colon (classified as severe disease type). Two other outcomes used to separate mild and severe disease type were number of bowel resections and the percentage of years in which a patient received systemic treatment (glucocorticoids, thiopurines, methotrexate or biologicals). Both the number of bowel resections as wells as the number of years in which a patient received systemic treatment were corrected for disease duration to exclude bias by exposure time.

### Cytokine measurement

Cytokines were measured in whole blood cultures after stimulation with *E*.*coli* O55:B5 LPS (Sigma Chemical, St. Louis, USA). Compared to isolated peripheral blood mononuclear cells (PBMCs), measuring cytokines in a whole blood culture has the advantage of preserving the physiological proportions of all natural components of the blood [34]. Briefly, heparinized blood samples (BD Vacutainer, BD, Belliver Industrial Estate, Plymouth, United Kingdom) were drawn and within one hour of collection 2 ml blood was diluted 1:5 with RPMI 1640 (RPMI; PAA Laboratories GmbH) supplemented with glutamine. Diluted blood was seeded into a 6-well microtiter plate and stimulated with 1 ng/ml (final concentration) LPS. Blood was incubated at 37°C and 5% CO_2_. Supernatants were harvested after four hours (TNF-α) and 24 hours (other cytokines) of incubation and stored at -80°C until usage [28]. TNF-α levels were measured using specific Pelikine compactTM human TNF-α enzyme-linked immunosorbent assay (ELISA) kits (Sanquin, Amsterdam, The Netherlands) according to the manufactures instructions with a sensitivity of 1–3 pg/ml. IL-1β, IL-6 and IL-10 were measured with specific Duoset ELISA Development kits (R&D Systems Inc, Minneapolis, MN, USA), with a sensitivity of 3.91 pg/mL for IL-1β (http://www.rndsystems.com/Products/DY201-05), 9.38 pg/mL for IL-6, (http://www.rndsystems.com/Products/dy206-05/), and 31.2 pg/ml for IL-10 (http://www.rndsystems.com/Products/DY217B-05), respectively. Cytokine levels where corrected for total leukocyte counts.

### Polymorphisms in the TNF-α promoter region

The presence of single nucleotide polymorphisms (SNPs) in the promoter region of *TNF-α* has been linked to the TNF-α production [12] and is in this way used as a surrogate marker to explore correlations between TNF-α production and disease characteristics [35]. To test this assumption in our cohort we correlated SNPs in the promoter region of the *TNF-α* gene with TNF-α levels. Herefore, genomic DNA was isolated with the High Pure PCR Template Preparation Kit (Roche Diagnostics GmbH, Mannheim, Germany) according to the manufacturers protocol. This kit removes heparin from the sample and therewith avoids its influence on the polymerase chain reaction (PCR). After dividing it into two blocks the *TNF-α* promoter region was amplified by PCR using the primers depicted in Table 1. PCR products were analyzed by Sanger sequencing on an ABI3730 Genetic Analyzer (Applied Biosystems). A combination of Gene Runner, version 3.00 (Hasting Software, Inc) and ChromasPro, version 1.15 (Technelysium Pty Ltd, Australia) was used to screen the *TNF-α* promoter region for the presence of SNPs. Analysis were done prior to measurement of TNF-α levels.

**Table 1:** Primer sequences and conditions for the TNF-α promoter gene.

	Primer	Sequence	Temperature	[Mg]
Block 1	Forward	5’-CTGTGGGGAGAACAAAAGGAT	56°C	2.0 mM
	Reverse	5’-GGACCAGGTCTGTGGTCTGT		
Block 2	Forward	5’-AACACAGCTTTTCCCTCCAA	62°C	3.0 mM
	Reverse	5’-CAGCTTGTCAGGGGATGTG		

Mg_:_ Magnesium concentration.

### Statistics

Depending on the distribution of the data, mean with SD (parametric) or median with IQR (non-parametric) test were used. The relation between SNPs in the promoter region of the *TNF-α* gene and TNF-α production was analyzed using the Kruskal-Wallis test. Haplotypes of polymorphisms in the *TNF-α* promoter region seen in more than three patients were tested for associations with TNF-α production. A two-tailed p-value of <0.05 was considered significant and Bonferroni correction was used to deal with multiple testing. Statistical analysis was performed by using SPSS version 20.0.0.1 (SPSS Inc, Chicago, IL, USA).

## Results

### Baseline characteristics

In total, 75 patients with histologically confirmed CD were included. Six patients were excluded for analysis based on elevated CRP levels or leukocyte counts. Baseline characteristics of the remaining 69 patients and 24 healthy controls are depicted in Table 2. There was no significant difference in gender (p = 0.97) and age (p = 0.2) between patients and controls.

**Table 2:** Baseline characteristics of the included patients with CD and HC.

	CD (n = 69)	HC (n = 24)
Female, n (%)	50 (72.5)	18 (75.0)
Age, mean years (SD)	51.2 (12.3)	54.7 (9.5)
Disease duration, mean years (SD)	24.1 (11.5)	-
**Age at diagnosis, n (%)**		
A1 (<17 years)	5 (7.2)	-
A2 (17–40 years)	57 (82.6)	-
A3 (>40 years)	7 (10.1)	-
**Disease localization, n (%)**		
L1 (ileal disease)	33 (47.8)	-
L2 (colonic disease)	8 (11.6)	-
L3 (ileocolonic)	28 (40.6)	-
L4 (Upper GI-tract)	1 (1.4)	-
Perianal disease	21 (30.4)	-
**Prior disease behavior, n (%)**		
B1 (non stricturing, non fistulating)	14 (20.3)	-
B2 (stricturing)	44 (63.8)	-
B3 (fistulating)	38 (55.1)	-
**Current 5-ASA use, n (%)**	17 (24.6)	0 (0)
**Prior drug history, n (%)**		
Steroids	62 (89.9)	0 (0)
Thiopurines	37 (53.6)	0 (0)
Biologicals	6 (8.7)	0 (0)
Methotrexate	1 (1.4)	0 (0)
Cyclosporine	2 (2.9)	0 (0)
**Prior extra-intestinal manifestations**		
Arthralgia, n (%)	18 (26.1)	-
**Number of bowel resections in the past, n (%)**		
1	48 (69.6)	0 (0)
2	16 (23.2)	0 (0)
≥ 3	5 (7.2)	0 (0)
**Mild versus severe disease, n (%)**		
Mild disease (L1 without history of fistulas)	27 (39.1)	-
Severe disease (L3 with history of fistulas)their disease course we are not afraid that this might influence	16 (23.2)	-

CD, Crohn’s disease; HC, healthy controls; SD, standard deviation; 5-ASA, 5-aminosalicylic acid.

### Healthy controls versus patients with quiescent CD

Patients with CD had significant higher TNF-α levels upon stimulation with LPS (p = 0.02). No difference was seen with IL-1β, IL-6 and IL-10 between patients with CD and healthy controls (Table 3).

**Table 3:** cytokine production in healthy controls versus patients with quiescent CD.

	Healthy controls (n = 24)	CD (n = 69)	*P* value
TNF-α [IQR]	428 [267–468]	459 [364–570]	0.02
IL-1β [IQR]	679 [565–878]	561 [374–850]	0.11
IL-6 [IQR]	2687 [1830–3228]	2384 [1703–3049]	0.39
IL-10 [IQR]	80 [61–149]	82 [59–107]	0.52

Cytokine levels (pg/ml) are presented as medians with the interquartile range [IQR]. TNF-α, Tumor necrosis factor alpha; IL, Interleukin; IQR, interquartile range; CD, Crohn's disease.

#### Correlation between cytokine production and disease phenotypes

**Fig 1:**
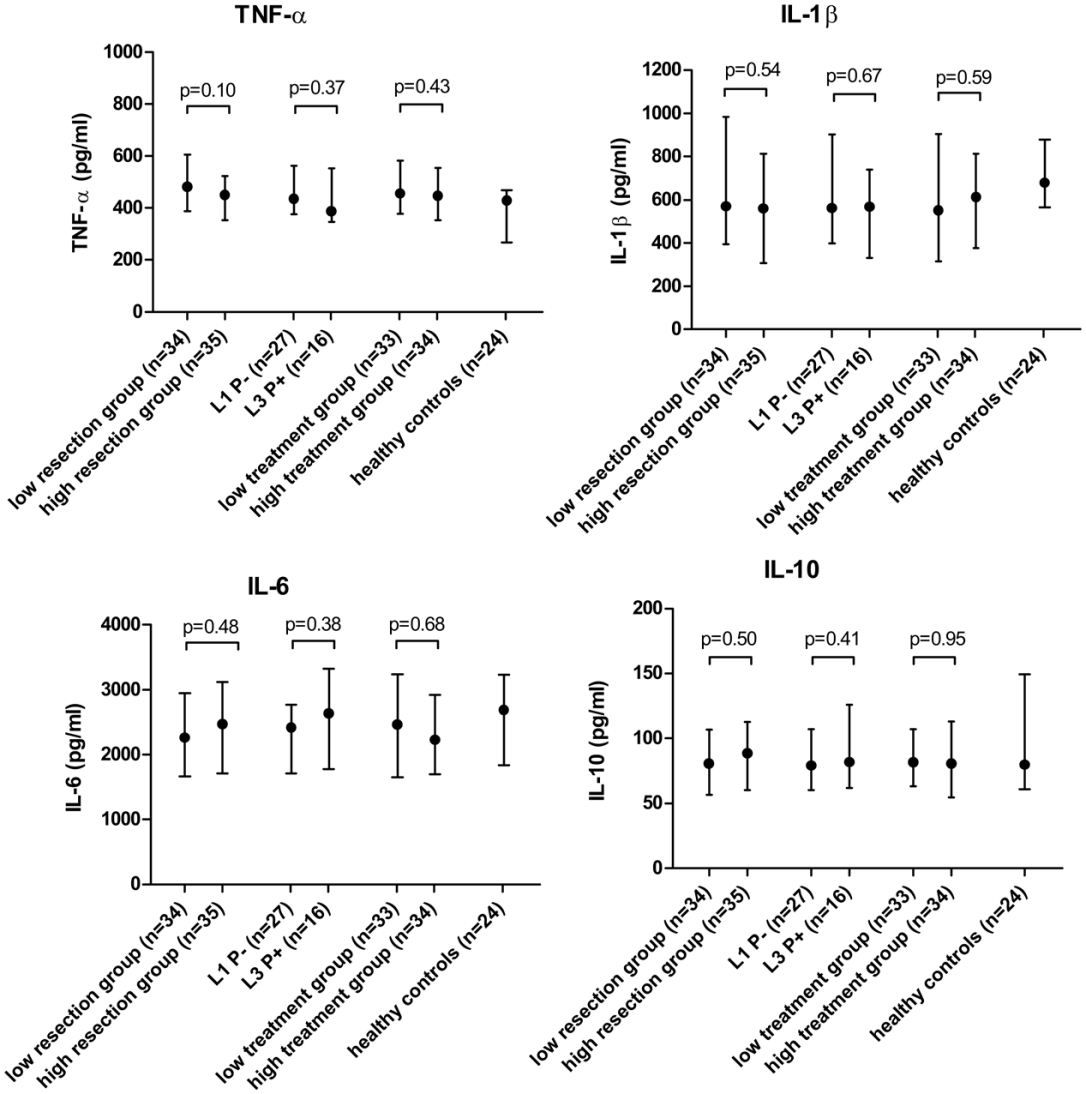
Relation between cytokine production and disease severity. Values are presented as medians (pg/ml) with the interquartile range. For the number of bowel resections and treatment exposure patients were dichotomized in a low and high group according to the number of resections or the percentage of years in which they were treated with steroids, thiopurines, biologicals, methotrexate or cyclosporine, corrected for disease duration. Two patients were excluded for the analysis for treatment exposure, because no complete drug history was available. TNF-α, Tumor necrosis factor alpha; IL, Interleukin; L1, ileal disease; L3, ileocolonic disease; P-, no fistulas; P+, fistulating disease.

Table 4 and Fig 1 show the median cytokine production and corresponding p-values in relation to disease characteristics and disease severity. None of the disease characteristics showed a significant correlation with TNF-α, IL-1β, IL-6 or IL-10 levels.

**Table 4:** Cytokine production and disease characteristics.

	TNF-α [IQR]	*P* value	IL-1β [IQR]	*P* value	IL-6 [IQR]	*P* value	IL-10 [IQR]	*P* value
HC (n = 24)	428 [267–468]		679 [565–878]		2687 [1830–3228]		80 [61–149]	
**Localization**								
L1 (n = 33)	454 [362–624]		561 [356–903]		2384 [1585–3017]		89 [65–107]	
L2 (n = 8)	516 [469–560]		603 [237–1243]		2715 [1701–3465]		64 [30–83]	
L3 (n = 28)	421 [354–561]	0.41	568 [387–750]	0.90	2395 [1756–2830]	0.79	81 [55–112]	0.22
P—(n = 48)	452 [368–573]		569 [380–901]		2384 [1711–2872]		88 [64–111]	
P + (n = 21)	483 [325–575]	0.98	560 [272–747]	0.58	2491 [1469–3332]	0.92	75 [55–107]	0.32
**Behavior**								
No fistulas (n = 31)	454 [400–563]		561 [384–905]		2417 [1707–2816]		79 [55–107]	
Fistulas (n = 38)	462 [350–579]	0.55	568 [298–813]	0.62	2351 [1567–3305]	0.81	82 [62–108]	0.50
No strictures (= 25)	408 [351–584]		511 [254–859]		2402 [1691–3062]		90 [67–114]	
Strictures (n = 44)	476 [387–573]	0.38	569 [400–866]	0.45	2384 [1703–3049]	0.92	77 [54–107]	0.12
**Extra intestinal manifestations**								
No arthralgia (n = 51)	454 [373–578]		577 [379–902]		2445 [1706–3045]		85 [62–106]	
Arthralgia (n = 18)	476 [348–562]	0.90	550 [261–796]	0.51	2204 [1588–3088]	0.46	82 [53–124]	0.77

Cytokine levels (pg/ml) are presented as medians with the interquartile range [IQR]. HC, Healthy control; TNF-α, Tumor necrosis factor alpha; IL, Interleukin; L1, ileal disease; L2, colonic disease; L3, ileocolonic disease; P-, no peri-anal disease; P+, peri-anal disease; IQR, interquartile range.

### Polymorphisms in the TNF-α promoter region and TNF-α production

**Fig 2:**
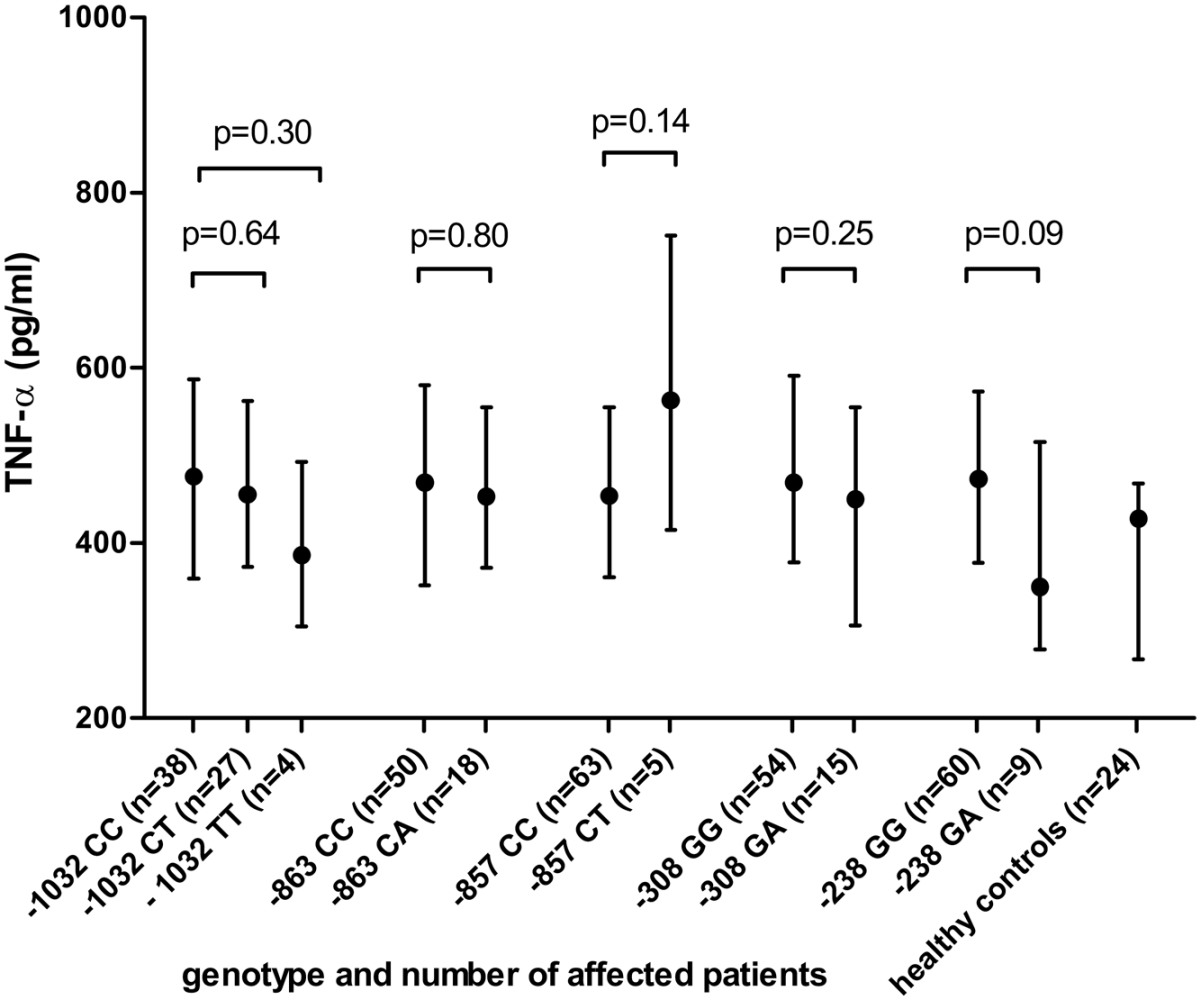
TNF-α production (pg/ml) according to (SNPs) in the promoter region of the *TNF-α* gene. Values are expressed as medians with the interquartile range. The median value of the healthy controls serves as reference. TNF-α, Tumor necrosis factor alpha; SNPs, single nucleotide polymorphisms

Fig 2 shows the TNF-α values of patients with various genotypes in the *TNF-α* promoter region. We found no evidence for an increased TNF-α production in LPS-stimulated whole blood cultures of patients with one or more SNPs in the *TNF-α* promoter region. The -308A genotype, the most used surrogate marker for a high producer phenotype, showed a median [IQR] TNF-α production of 450 pg/ml [306–554] compared to 469 pg/ml [378–590] in patients with the most common genotype (P = 0.25). The -863AA genotype (TNF-α production of 523 pg/ml) and -857TT genotype (TNF-α production of 1053 pg/ml) were only detected once and were not included in Fig 2. The haplotypes, -1032T with -863A (n = 12), and -1032T with -238A (n = 4) were not related with TNF-α production.

## Discussion

We found that the inter individual variation in LPS-induced production of TNF-α, IL-1β, IL-6 and IL-10 in whole blood cultures was not related to disease phenotype in patients with quiescent CD who were not taking immunomodulators or biologicals. Also, when disease characteristics where combined to differentiate mild from more severe disease type no differences were found.

The innate cytokine production by an individual is constant over time, but might be affected by many variables such as disease activity and concomitant drug treatment [16,22–29,31,36]. For this reason we excluded patients with disease activity or maintenance therapy, thereby minimizing variables other than genetic that can modulate cytokine production as far as possible. To our knowledge, only a limited number of studies included patients with CD in remission without maintenance therapy [5,21,37]. In these studies decreased levels of TNF-α and IL-6 produced by monocyte derived macrophages compared to healthy controls were found. Furthermore, an association with disease localisation in patients with CD was seen e.g. patients with colonic disease (L2) had significant lower TNF-α production upon LPS stimulation compared to patients with only ileal disease (L1) [21]. We did not find a significant difference in cytokine production between patients with L1 and L2 disease in our study. A possible explanation here might be that in our cohort only a small number of patients (n = 8; 11.6%) had exclusively colonic disease, compared to 44 (43.6%) in the study of Sewell et al. [21] Another explanation might be that they studied cytokine production from monocyte-derived macrophages, while we studied whole blood cultures. To our knowledge there are no studies comparing cytokine production of whole blood cultures (including all natural components) with a homogeneous cell suspension of monocyte-derived macrophages, which makes it difficult to interpret these differences. It is unlikely that the higher TNF-α levels which we found in patients with CD were caused by disease activity, but this cannot completely be excluded. A more likely explanation can be found in the different cell populations being investigated. The presence of neutrophils in a whole blood culture is the most important difference in experimental setup compared to stimulation of isolated peripheral monocytes or monocyte derived macrophages. Since TNF-α production is equal in isolated peripheral monocytes in CD and healthy controls [38], it might be that an impaired inhibitory function by neutrophils on pro-inflammatory cytokine production led to higher TNF-α values in patients with CD [39]. A recent study indeed showed a disturbed cell signaling in neutrophils from patients with CD, which supports our hypothesis [40]. However, further research is warranted to elucidate this mechanism.

With respect to IL-6, there is another study which also used LPS stimulated whole blood cultures and measured IL-6 levels in patients with CD and HD. They found reduced IL-6 levels in patients with CD, however this study included patients with active disease and on maintenance therapy [41]. This might have influenced IL-6 levels and such makes it hard to compare with our results.

IL-10 is considered the most potent anti-inflammatory cytokine in the human immune response, playing a key role in immune homeostasis. Its significance for CD follows from IL-10 knock-out mice developing CD-like enterocolitis [42]. Furthermore, SNPs in IL-10 may predispose for young onset CD, which is considered as more severe and difficult to treat [43]. Therefore, it can be hypothesized that patients with a high producer phenotype of IL-10 have a milder disease course [19]. A study in patients with CD in remission showed that IL-10 levels were decreased in patients with a history of fistulating disease [44]. An important difference with our study is that the majority of the patients in the latter study used immunomodulators. Since fistulas are more difficult to treat, it is likely that especially these patients were on immunosuppressive medication. Knowing that immunomodulating agents affect cytokine production, this might have influenced their findings [23].

Techniques of measuring cytokine production in peripheral blood have been well established. However, apart from studies comparing cytokine levels of healthy persons with patients with CD, the application of these techniques to explore a relation with disease characteristics in CD is limited to a few recent studies [20,21,44]. An alternative strategy to categorize patients as high- or low producers is to look at SNPs involved in cytokine regulation. These SNPs can lead to an increase or decrease in cytokine production and their presence is therefore used as a surrogate marker for high- or low producer phenotype [35,45]. Our study was not primarily designed to explore an association between SNPs in the *TNF-α* gene and TNF-α production. Even so the results do not support such a relation, which is in line with recent literature [46]. Therefore, it seems more appropriate to categorize patients as being low or high producer based on actual cytokine measurements rather than on genetic proxy’s.

Our study comes with some limitations. First of all, the power of our study to pick up more subtle associations can be compromised by our strict inclusion criteria, which limited the number of enrolled patients. Nevertheless, the included patients represent a unique population of patients with CD in whom immune functions are probably not disturbed by disease activity or concomitant use of immunomodulating drugs. Furthermore, we defined “remission” as a Harvey-Bradshaw Index of ≤ 4, in combination with normal leukocyte counts and CRP levels. Although colonoscopy is the golden standard to exclude mucosal inflammation, this was considered a too high burden for our patients in this setting. The strict inclusion criteria precluded participation of patients with maintenance therapy or active disease, i.e. those with a potentially severe disease course. However, compared to a previous study in patients with CD in our hospital no differences were seen in disease characteristics [47], suggesting that our inclusion criteria did not lead to significant selection bias. Differences in the moment of diagnosis may have influenced the outcomes for drug exposure to some extent, because of evolving treatment regimes. More specifically, patients with a longer disease history were relatively underexposed to new treatment modalities, while patients with a more recent date of diagnosis usually are confronted with steroid-free treatment strategies. To prevent bias, we merged the data of glucocorticoid, thiopurine, biological and other immunosuppressants use. This gave us a valuable impression for the need of immunnosupressive drugs to control disease activity. In order to prevent bias, medical records and presence of SNPs in the TNF-α promoter region were analyzed before measuring TNF-α production.

In conclusion, in this study TNF-α, IL-1β, IL-6 and IL-10 production in LPS-stimulated whole blood cultures of patients with CD in remission and without taking immunomodulating drugs or biologicals could not be correlated to historical disease phenotype, course or severity. This suggests that individual differences in innate cytokine production are not important for the development of disease phenotype in CD.
